# Complications and mortality after catheter ablation of ventricular arrhythmias: risk in VT ablation (RIVA) score

**DOI:** 10.1007/s00392-021-01902-2

**Published:** 2021-07-27

**Authors:** Shibu Mathew, Thomas Fink, Sebastian Feickert, Osamu Inaba, Naotaka Hashiguchi, Michael Schlüter, Peter Wohlmuth, Erik Wissner, Roland Richard Tilz, Christian-Hendrik Heeger, Laura Rottner, Bruno Reissmann, Andreas Rillig, Andreas Metzner, Tilman Maurer, Karl-Heinz Kuck, Feifan Ouyang

**Affiliations:** 1 Department of Cardiology, Asklepios Klinik St. Georg, Hamburg, Germany; 2 Department of Cardiology, University of Giessen, University Hospital Giessen, Giessen, Germany; 3 Clinic for Electrophysiology, Herz- und Diabeteszentrum Nordrhein-Westfalen, Bad Oeynhausen, Germany; 4 LANS Cardio, Hamburg, Germany; 5 Asklepios Proresearch, Hamburg, Germany; 6 College of Medicine, University of Illinois Chicago, Chicago, IL USA; 7 Department of Electrophysiology, University Heart Center Lübeck, Medical Clinic II, University Hospital Schleswig-Holstein, Lübeck, Germany; 8 Present Address: University Heart Center Hamburg, Hamburg, Germany

**Keywords:** Catheter ablation, Ventricular tachycardia, Epicardial, Risk score, Mortality

## Abstract

**Aims:**

Catheter ablation of ventricular arrhythmias (VA) has proven to be an effective therapeutic option for secondary arrhythmia prophylaxis. We sought to assess the procedural efficacy, safety and in-hospital mortality of a large patient cohort with and without structural heart disease undergoing VA ablation.

**Methods:**

A total of 1417 patients (804 patients with structural heart disease) undergoing 1792 endo- and epicardial procedures were analyzed. Multivariable risk factor analysis for occurrence of major complications and intrahospital mortality was obtained and a score to allow preprocedural risk assessment for patients undergoing VA ablation procedures was established.

**Results:**

Major complication occurred in 4.4% of all procedures and significantly more often in patients with structural heart disease than in structurally normal hearts (6.0 vs. 1.8%). The frequency of these periprocedural complications was significantly different between procedures with sole right ventricular and a combination of RV and LV access (0.5 vs. 3.1%). The most common complication was cardiac tamponade in 46 cases (3.0%). Intrahospital death was observed in 32 patients (1.8%). Logistic regression model revealed presence of ischemic heart disease, epicardial ablation, presence of oral anticoagulation or dual antiplatelet therapy as independent risk factors for the occurrence of complications or intrahospital death, while a history of previous heart surgery was an independent predictor with a decreased risk. Based on this analysis a risk score incorporating 5 standard variables was established to predict the occurrence of complications and intrahospital mortality.

**Conclusions:**

Safety of VA catheter ablation mainly relies on patient baseline characteristics and the type of access into the ventricles or epicardial space.

**Supplementary Information:**

The online version contains supplementary material available at 10.1007/s00392-021-01902-2.

## Introduction

Catheter ablation of ventricular arrhythmias (VA) has proven to be an effective therapeutic option for secondary arrhythmia prophylaxis [1–4]. Recent ablation modalities diverge from pure endocardial approaches to combined epi- and endocardial mapping and ablation strategies. Earlier studies suggested a correlation between structural heart disease (SHD) and other baseline parameters with the occurrence of procedural complications [5, 6]. Additionally, procedures with epicardial access are known to bear a high potential risk for intraprocedural complications [7, 8]. Available data on large patient cohorts evaluating the procedural characteristics of VA outside of the USA are sparse and are mainly reported on VA ablation in patients with SHD [5, 6].

In this study we assessed the procedural efficacy, safety and in-hospital mortality of a large patient cohort undergoing VA ablation for idiopathic or structural VA in a high-volume electrophysiology center. Our goal was to determine predictive factors for the occurrence of periprocedural complications analyzing clinical and procedural parameters. Subsequently, we developed a score using basic parameters to allow preprocedural risk assessment for patients undergoing VA ablation procedures.

## Methods

### Study design and patient inclusion

This study included patients undergoing VA ablation from 2002 to 2017 in the Asklepios Klinik St. Georg, Hamburg, Germany. The analysis was approved by the local ethics committee (Processing Number: WF-48/17). All patients included in the analysis gave written informed consent to the ablation procedure and patient information was anonymized for analysis. The patient cohort consisted of patients with idiopathic VA and of patients with structural heart disease. The type of underlying heart disease was classified as structurally normal hearts, ischemic cardiomyopathy (iCMP), dilated cardiomyopathy (dCMP), arrhythmogenic right ventricular cardiomyopathy (ARVC) and other cardiomyopathies (including valvular heart disease, congenital heart disease and patients with a history of myocarditis).

Indication for VA ablation was derived from corresponding guidelines. PVC ablation was conducted in patients with clinical symptoms due to a high PVC burden, excercise induced PVCs or had high PVC burden (usually at least  >  10%) or impaired LV function associated with presence of PVC. Indication of ablation of sustained VA depended on underlying heart disease (e.g., ischemic disease, dilated cardiomyopathy), presence and number of ICD shocks, presence of suitable ablation targets and clinical symptoms.

### Procedural set up

Procedures for sustained VA were conducted under deep sedation using fentanyl or sufentanyl, midazolam and propofol under continuous monitoring of vital parameters. Procedures were performed under general anesthesia with endotracheal intubation only in patients with decompensated heart failure, hemodynamically instable VT/electrical storm, or impaired oxygenation. In patients admitted for PVC ablation sedation was administered if PVCs were present during baseline conditions and during procedure. Additionally, provocation maneuvers like isoproterenol infusion and intracardiac stimulation were performed. If PVCs cessated during deep sedation patients were treated without sedation.

Transthoracic echocardiography with or without contrast medium was performed before the procedure in patients without known atrial fibrillation (AF) to assess left ventricular ejection fraction (LV-EF) and to rule out left ventricular (LV) thrombi. Transesophageal echocardiography (TEE) was performed to rule out intracardiac thrombi before transseptal puncture, which was used as an antegrade approach into the left ventricle, in case of underlying AF and anticipated left ventricular origin of the VA. After transseptal puncture intravenous heparin was administered targeting an activated clotting time (ACT)  >  300 s.

In cases of preexisting treatment with vitamin K antagonists, the procedure was performed on bridging therapy with low-molecular heparin (LMH) and held  >  6 h before procedure until 2012. After 2012 no bridging was performed and vitamin K antagonists were not interrupted aiming INR values in therapeutic ranges at the day of the procedure. Novel oral anticoagulant (NOAC), were discontinued 24 h before the procedure, if medically acceptable. Anti-arrhythmic drug therapy (AAD) except amiodarone were discontinued at least 5 half-times before the procedure, if possible.

### Electrophysiological study, electroanatomical mapping and ablation

Two diagnostic catheters were introduced via the right femoral vein or subclavian vein and positioned in the coronary sinus (CS) and the right ventricle (RV). Subclavian access was used on operators discretion to obtain access to the CS. The stimulation protocol consisted of programmed stimulation from the RV at two drive cycle lengths (CL 510 and 440 ms) with up to three extrastimuli to a minimum coupling interval of 200 ms. Burst-pacing with the shortest CL of 250 ms was also used if induction failed with programmed stimulation.

Mapping was performed using a 7F 3.5 mm tip (Navistar Thermocool^®^, Biosense Webster Inc., Diamond Bar, CA, USA) through the femoral vein under fluoroscopic guidance. Our methods were previously described in detail [9]. In brief, 3D electroanatomic maps (EAM) were performed during sinus rhythm (SR) and/or during VT in case of stable and reproducible VT. Activation and voltage maps were both used to guide the ablation strategy. In patients with structurally normal hearts, activation mapping and pacemapping were used to guide ablation. In patients with structural heart disease, ablation was performed at the site of diastolic potentials during VT in case of stable VT and short post pacing intervals after entrainment of the VT or guided by pace mapping at the substrate area with fractionated or late potentials. Fragmented low voltage signals or late potentials that were judged to be crucial to maintain the induced VT circuit were targeted for ablation. If no sustained VT was inducible, all fragmented low voltage signals/late potentials in the region where the documented clinical VA were suspected were eliminated. VT ablation strategies contained ablation of the clinical VT (if ECG and/or electrogram was available) as well as mapping and ablation of late potentials according to cardiac mapping. Achievement of late potential elimination was based on operators’ judgment. Irrigated radio-frequency current was delivered in power-controlled mode, power 30–40 W, irrigation 17–30 ml/min, and temperature limit 43 °C.

Epicardial access was obtained through a subxiphoid puncture. Dry epicardial puncture was obtained under fluoroscopic guidance in antero-posterior or left anterior obqlique (60°) views. Puncture needles varied during the study period and conventional puncture needles as well as micro puncture needles were used. Heparin was withheld until successful epicardial access was reached. After reaching the pericardial space, contrast agent was injected to verify the position of the needle in the pericardial space, and a long J-tipped guidewire was placed via the needle in the pericardial space. After positioning a long 8, 5 Fr SL-1 sheath in the pericardial space over the guidewire, the ablation catheter was advanced into the pericardial space and ablation energy was delivered applying a power of 30–40 W, flush rate 17–20 ml/min and maximum temperature of 43 °C. Repeat aspiration of irrigation fluid was performed depending on hemodynamic status or after every fifth radio-frequency application within the epicardial space.

The procedural endpoint was non-inducibility of any sustained VT by programmed stimulation  ±  isoproterenol at the end of the procedure (complete success). Partial success was defined as non-inducibility of clinical VT, but remaining inducibility of non-clinical sustained VT or fast VT (CL  <  240 ms). In case of lack of VT inducibility prior to ablation, the procedural endpoint was attempted to eliminate all fragmented low voltage signals/late potentials in the region where the documented clinical VA were suspected. Acute ablation failure was defined if clinical VT remained inducible. Post-procedural in-hospital ablation success was evaluated with Holter ECG recordings at the first post-procedural day, daily 12-lead ECG recordings, interrogations of intracardiac devices (if available) and continuous telemetry monitoring on hospital wards in patients with sustained VA.

### Post-procedural care and in-hospital follow-up

All patients underwent transthoracic echocardiography immediately and the day after the procedure to rule out pericardial effusion. After epicardial mapping/ablation the epicardial pigtail catheter was removed after exclusion of pericardial effusion. A chest X-ray to rule out pneumothorax was performed in patients with attempted subclavian vein access. Preexisting therapy with Vitamin K antagonists was continued aiming at an INR of 2.0–3.0. NOACs were reinitiated earliest 6 h after the procedure.

### Definition of periprocedural complications

Periprocedural complications were defined as major when being life-threatening or resulting in patient death, temporal (at least until discharge) or permanent patient disability, leading to percutaneous or surgical intervention, leading to transfusion of blood products or leading to prolonged hospitalization. Other complications which resulted in pain, discomfort or further diagnostic procedures were categorized to be minor complications. All complications occurring during the hospital stay were analyzed. Patient data was collected until discharge and all adverse events and deaths were documented.

### Statistics

The primary aim of this retrospective analysis was the development of a scoring system to calculate the risk of potential complications during or after ablation of VA. The target was severe complications including tamponade and mortality. The dataset consisted of 1792 patients. Demographic data, risk factors and complications data were recorded. Continuous data were summarized as means  ±  standard deviations or as medians [25th and 75th percentiles] as appropriate. Categorical data were presented as N (%). Differences between continuous data were calculated with the student’s *t *test without correction for numbers of conducted tests. Differences between categorical data were analyzed with the Chi square or Fisher’s exact test, as appropriate. A logistic regression model was applied to predict complications from risk factors and procedural data. Redundancy analyses were used to reduce the number of variables. Missing values were imputed beforehand. Goodness of fit statistics (explained variation) were used to select the final set of covariables. Additional analysis encompassed the effects of age, glomerular filtration rate (GFR) and anticoagulation on mortality using a logistic regression model. Both models were shown with parameter estimates and standard errors, p values, odds ratios (95% confidence intervals). A nomogram was presented to predict the probability of complications for further patients. All *p *values were two-sided and a *p *value  <  0.05 was considered significant. All calculations were performed with the statistical analysis software R (R Core Team, 2018).

## Results

### Patients baseline data

A total of 1417 patients [965 male, (68.1%)], mean age 64.4  ±  16.0 years) were analyzed. The underlying arrhythmia was ventricular tachycardia (VT) in 642 patients (45.3%), premature ventricular complexes in 610 patients (43.1%) and electrical storm in 165 patients (11.6%). In 613 patients (43.3%) no underlying heart disease was present, while 804 patients had underlying SHD with 474/1417 patients (33.5%) suffering from iCMP, 174/1417 from dCMP (12.3%), 52/1417 patients from ARVC (3.7%) and 104/1417 patients suffering from other SHD (7.3%). Mean LV-EF was 49.3  ±  15.7%. A total of 294 patients (20.8%) had at least one ICD shock before the procedure [6/613 (1.0%) in patients without SHD vs. 288 (35.8%) in patients with SHD, *p*  =  0.0001]. Detailed baseline data are depicted in Table 1.

**Table 1:** Baseline patient characteristics

	All	No SHD	With SHD	*p* value
*N* pts	1417	613	804	
*N* male pts	965 (68.1)	311 (50.7)	654 (81.3)	**< 0.0001**
Mean age (years)	64.4 ± 16.0	58.0 ± 16.2	69.3 ± 14.0	**< 0.0001**
Mean BMI (kg/m^2^)	27.0 ± 5.6	26.5 ± 6.7	27.4 ± 4.7	**0.003**
Mean LV-EF (%)	49.3 ± 15.7	61.8 ± 6.0	39.8 ± 14.1	**< 0.0001**
*N* pts with LV-EF < 30%	208 (14.7)	0 (0)	208 (25.9)	**< 0.0001**
Mean LVEDD (mm)	55.4 ± 8.6	50.6 ± 4.8	59.4 ± 9.0	**< 0.0001**
Underlying heart disease				
None	613 (43.3)	613 (100)	0 (0)	**< 0.0001**
iCMP	474 (33.5)	0 (0)	474 (59.0)	**< 0.0001**
dCMP	174 (12.3)	0 (0)	174 (21.6)	**< 0.0001**
ARVC	52 (3.7)	0 (0)	52 (6.5)	**< 0.0001**
Other	104 (7.3)	0 (0)	104 (12.9)	**< 0.0001**
Mean GFR (ml/min)	75.7 ± 18.4	84.1 ± 11.8	69.2 ± 19.9	**< 0.0001**
*N* pts with VT	642 (45.3)	179 (29.2)	463 (57.9)	**< 0.0001**
*N* pts with PVC	610 (43.1)	431 (70.3)	179 (22.3)	**< 0.0001**
*N* pts with ES	165 (11.6)	3 (0.5)	162 (20.2)	**< 0.0001**
*N* pts with ICD shock	294 (20.8)	6 (1.0)	288 (35.8)	**< 0.0001**
*N* pts with previous heart surgery	206 (14.5)	7 (1.1)	199 (24.8)	**< 0.0001**
*N* pts with aHTN	866 (61.1)	216 (35.2)	650 (80.9)	**< 0.0001**
*N* pts with AF	293 (20.7)	56 (9.1)	237 (29.5)	**< 0.0001**
*N* pts with lung disease	137 (9.7)	32 (5.2)	105 (13.1)	**< 0.0001**
*N* pts with PAD	44 (3.1)	3 (0.5)	41 (5.1)	**< 0.0001**
*N* pts on OAC	323 (22.8)	42 (6.9)	281 (35.0)	**< 0.0001**
*N* pts on DAPT	105 (7.4)	0 (0)	105 (13.1)	**< 0.0001**
*N* pts on AAD class I	105 (7.4)	59 (9.6)	46 (5.7)	**0.0074**
*N* pts on AAD class III	329 (23.2)	27 (4.4)	302 (37.6)	**< 0.0001**

Bold value indicates statistically signficance *p* < 0.05

Values are mean  ±  standard deviation or *N* (%)

*SHD *structural heart disease; *iCMP *ischemic cardiomyopathy; *dCMP *dilated cardiomyopathy; *ARVC *arrhythmogenic right ventricular cardiomyopathy; *pts *patients; *BMI *body mass index; *LV-EF *left ventricular ejection fraction; *LVEDD *left ventricular end diastolic diameter; *AF *atrial fibrillation; *aHTN *arterial hypertension; *PAD *peripheral artery disease; *sVA *sustained ventricular arrhythmia; *nsVA *non-sustained ventricular arrhythmia; *PVC *premature ventricular contraction; *GFR *glomerular filtration rate; *ICD *implantable cardioverter-defibrillator; *ATP *antitachycardic pacing; *OAC *oral anticoagulation; *VKA *vitamin K antagonist; *NOAC *novel oral anticoagulant; *DAPT *dual antiplatelet therapy; *AAD *anti-arrhythmic drug

### Procedural characteristics

A total of 1792 procedures (717 procedures in patients without SHD and 1075 in patients with SHD) were conducted. Complete procedural success was documented in 1384 cases (77.2%), partial success in 151 cases (8.4%) and absence of procedural success in 257 procedures (14.3%).

Mean procedure duration was significantly longer with significantly higher mean fluoroscopy dosages in patients with SHD (178.4  ±  93.7 vs.138.6  ±  84.1, *p*  <  0.0001 and 2675.6  ±  20.0 vs. 1394.6  ±  11.5 cGy*cm^2^, *p*  <  0.0001). Patients without SHD had significantly more procedures performed with sole RV access (299 patients (41.7%) vs. 142 patients (13.2%), *p*  <  0.0001), while sole retrograde LV access (235 patients (32.8%) vs. 352 patients (32.7%), *p*  =  0.0099), combined ante- and retrograde LV access (151 patients (21.1%) vs. 401 patients (37.3%), *p*  <  0.0001), and epicardial access (41 patients (5.7%) vs. 230 patients (21.4%), p < 0.0001) was significantly more often used in patients with structural heart disease (Table 2). Additionally, procedures in patients with structural heart disease were more often conducted with endotracheal intubation [7 patients (1.0%) vs. 83 patients (7.7%), *p*  <  0.00001]. Complete success was more often reached in patients without SHD [590 patients (82.6%) vs. 794 patients (73.8%), *p*  =  0.0003].

**Table 2:** Procedural parameters

	All	No SHD	With SHD	*p* value
*N* procedures	1792	717 (40.0)	1075 (60.0)	
Mean procedure duration (min)	162.4 ± 92.1	138.6 ± 84.1	178.4 ± 93.7	**< 0.0001**
Mean fluoroscopy time (min)	19.7 ± 78.0	22.3 ± 118.5	18.0 ± 25.1	0.25
Mean fluroscopy dosage (cGy*cm^2^)	2153.2 ± 18.6	1394.6 ± 11.5	2675.6 ± 20.0	**< 0.0001**
*N* procedures with sole RV access	441 (24.6)	299 (41.7)	142 (13.2)	**< 0.0001**
*N* procedures with sole retrograde LV access	587 (32.8)	235 (32.8)	352 (32.7)	0.99
*N* procedures with sole antegrade LV access	191(10.7)	30 (4.2)	161 (15.0)	0.97
*N* procedures with antegrade + retrograde LV access	552 (30.8	151 (21.1)	401 (37.3)	**< 0.0001**
*N* procedures with epicardial access	271 (15.1)^a^	41 (5.7)^a^	230 (21.4)^a^	**< 0.0001**
*N* procedures with epicardial ablation	191 (10.7)	26 (3.6)	165 (15.4)	**< 0.0001**
*N* pprocedures with ITN	90 (5.0)	7 (1.0)	83 (7.7)	**< 0.0001**
*N* procedures with complete success	1384 (77.2)	590 (82.3)	794 (73.9)	**0.0003**
*N* procedures with partial success	151 (8.4)	34 (4.7)	117 (10.9)	**< 0.0001**
*N* procedures with no success	257 (14.3)	93 (13.0)	164 (15.3)	0.17

Bold value indicates statistically signficance *p* < 0.05

Values are mean  ±  standard deviation or *N* (%)

*RV *right ventricular; *LV *left ventricular; *ITN *intubation

^a^21/2/19 patients without endocardial LV access

### Frequency of periprocedural complications

**Fig. 1:**
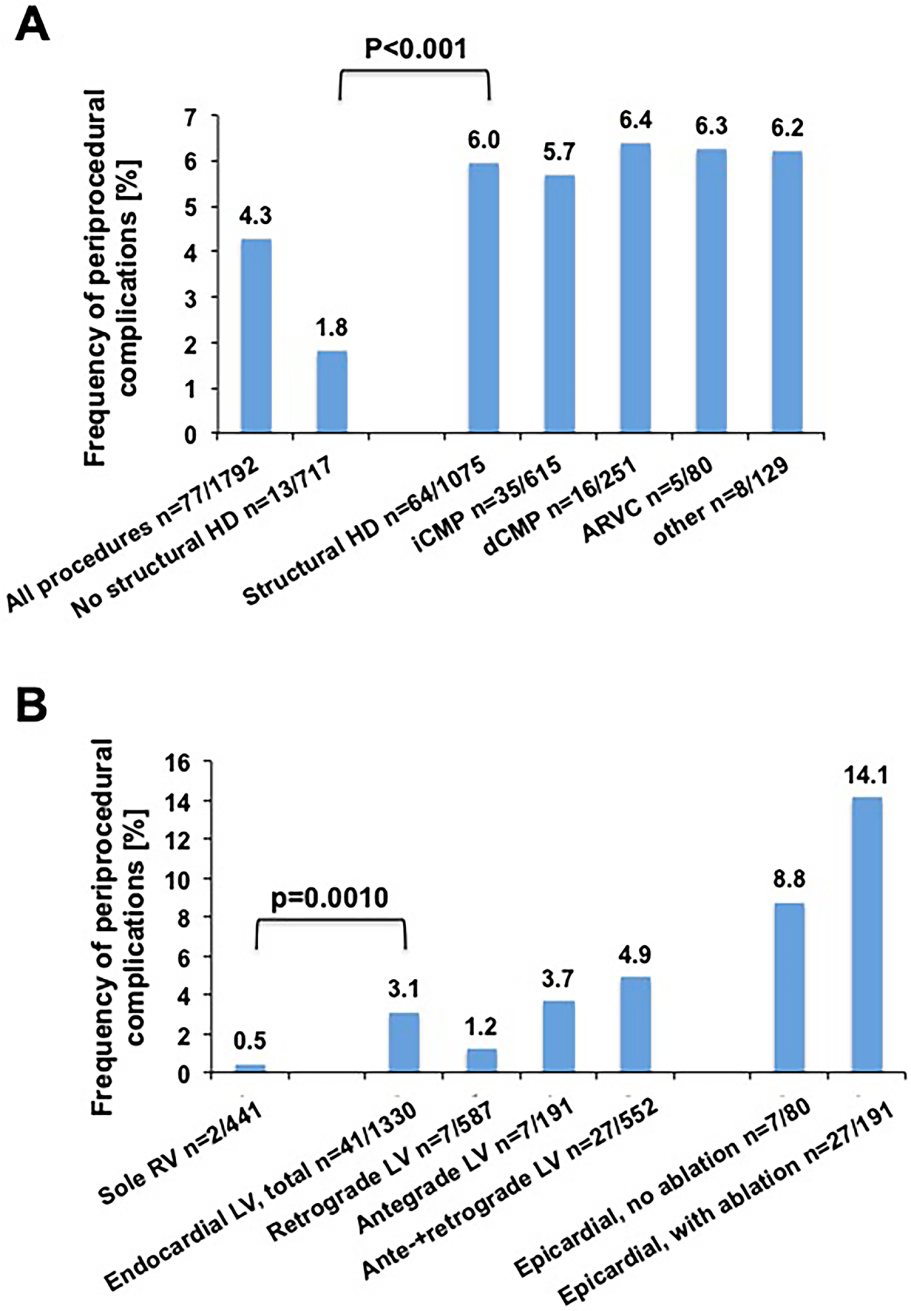
Frequency of major periprocedural complications. **A** Frequency of major periprocedural complications according to underlying heart disease. **B** Frequency of major periprocedural complications according to cardiac access type. *SHD* structural heart disease, *iCMP *ischemic cardiomyopathy, *dCMP *dilated cardiomyopathy, *ARVC *arrhythmogenic right ventricular cardiomyopathy, *RV *right ventricle, *LV *left ventricle

At least one major complication occurred in 77 procedures (4.3% of all procedures) and at least one minor complication occurred in 70 procedures (4.0%, Supplemental Table 1). Major complications occurred significantly more often in patients with SHD as compared to patients without SHD [13 patients (1.8%) vs. 64 patients (6.0%), *p*  <  0.001; Fig. 1A]. The most common major complication was cardiac tamponade, which occurred in 46 cases (3.0% of procedures). The frequency of major periprocedural complications was significantly different between procedures with pure RV access and use of LV access [2 patients (0.5%) vs. 41 patients (3.1%), *p*  =  0.001; Fig. 1B].

### Intrahospital mortality

**Fig. 2:**
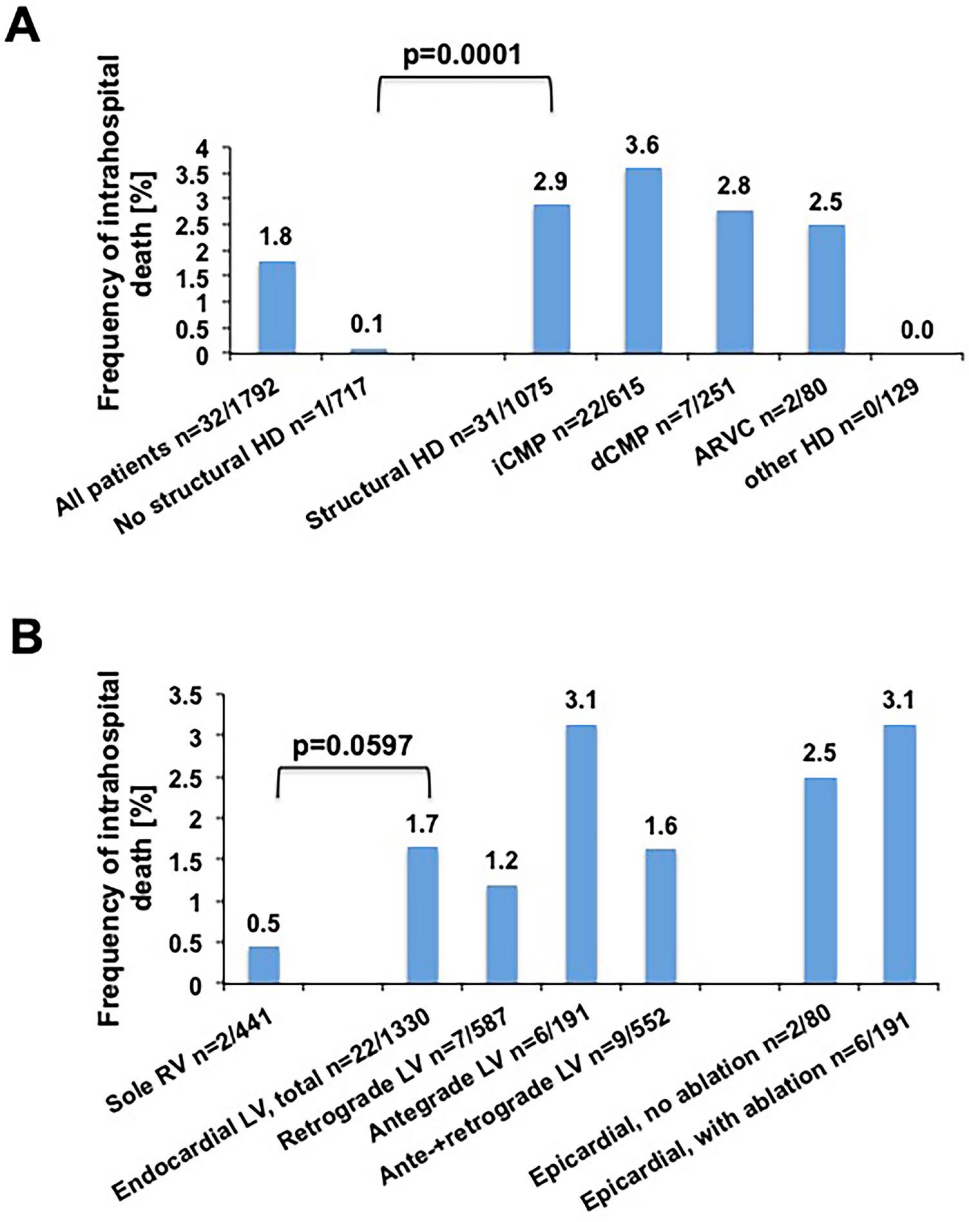
Frequency of intrahospital death. **A** Frequency of intrahospital death according to underlying heart disease. **B** Frequency of intrahospital death according to cardiac access type. *SHD *structural heart disease, *iCMP *ischemic cardiomyopathy, *dCMP *dilated cardiomyopathy, *ARVC *arrhythmogenic right ventricular cardiomyopathy, *RV *right ventricle, *LV *left ventricle

Figure 2A, B show the distribution of intrahospital deaths in relation to underlying heart disease (A) and applied access type (B). Intrahospital death was observed in 32 patients (1.8%). The frequency of intrahospital death was significantly lower in patients without SHD as compared to patients with SHD [1/717 patients (0.1%) vs. 31/1075 patients (2.9%), *p*  =  0.0001; Fig. 2A]. Intrahospital death occurred in 13/90 procedures (14.4%) with intubation and 19/1702 procedures (0.1%) without intubation.

**Fig. 3:**
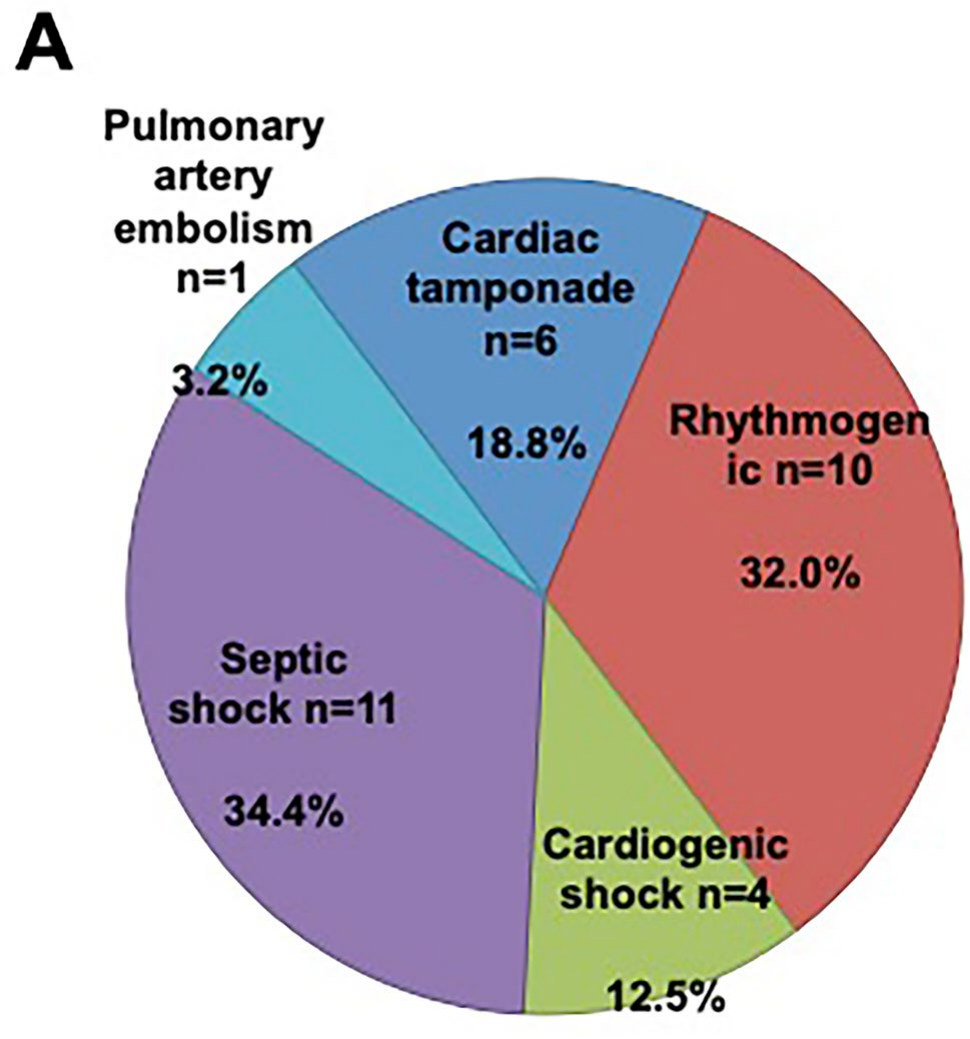
Cause of intrahospital death

Detailed reasons for intrahospital deaths are shown in Fig. 3. The most common causes were VA recurrence (10/32 patients, 32.0%) and sepsis (11/32 patients; 34.4%, 5 of these underwent VA ablation with endotracheal intubation). Another 6 patients (18.8%) died due to cardiac tamponade which occurred during VA ablation (5 patients with cardiac tamponade due to cardiac perforation and one patient with inappropriate transseptal puncture into the aortic root and subsequent surgical repair). Another 4 patients (12.5%) suffered from lethal cardiogenic shock after the procedure. One patient (3.2%) suffered from lethal pulmonary embolism.

### Risk factors for complications and intrahospital death and development of a predictive risk score

Logistic regression model revealed presence of iCMP, epicardial ablation, presence of either NOAC, VKA or dual antiplatelet therapy (DAPT) as independent risk factors for the occurrence of major complications or intrahospital death (Table 3). Presence of dCMP, ARVC and other SHD showed a trend towards positive prediction of intrahospital complications and intrahospital mortality. A history of previous heart surgery was an independent predictor of a lower rate of complications and death (Table 3).

**Table 3:** Logistic regression model of predictors of major complications and intrahospital death

	Estimate	Std error	Odds ratio	95% CI	*p* value
Ischemic CMP	1.190	0.365	3.29	1.61–6.72	**0.001**
Dilated CMP	0.753	0.405	2.12	0.96–4.69	0.063
ARVC	0.919	0.513	2.51	0.92–6.85	0.073
Other SHD	0.858	0.478	2.36	0.93–6.02	0.072
GFR	− 0.025	0.009	0.74	0.60–0.92	**0.009**
Epicardial ablation	1.189	0.254	5.4	2.00–5.40	**< 0.001**
NOAC/VKA/DAPT	0.526	0.247	2.74	1.04–2.74	**0.033**
Previous heart surgery	− 0.698	0.32	0.5	0.27–0.93	**0.029**

Bold value indicates statistically signficance *p* < 0.05

Variables are related to occurrence of major complication or intrahospital death

*Std error* standard error; *CI *confidence interval; *CMP *cardiomyopathy; *ARVC *arrhythmogenic right ventricular cardiomyopathy; *SHD *structural heart disease; *GFR *glomerular filtration rate; *NOAC *novel oral anticoagulation; *VKA *vitamin K antagonist; *DAPT* dual antiplatelet therapy

**Fig. 4:**
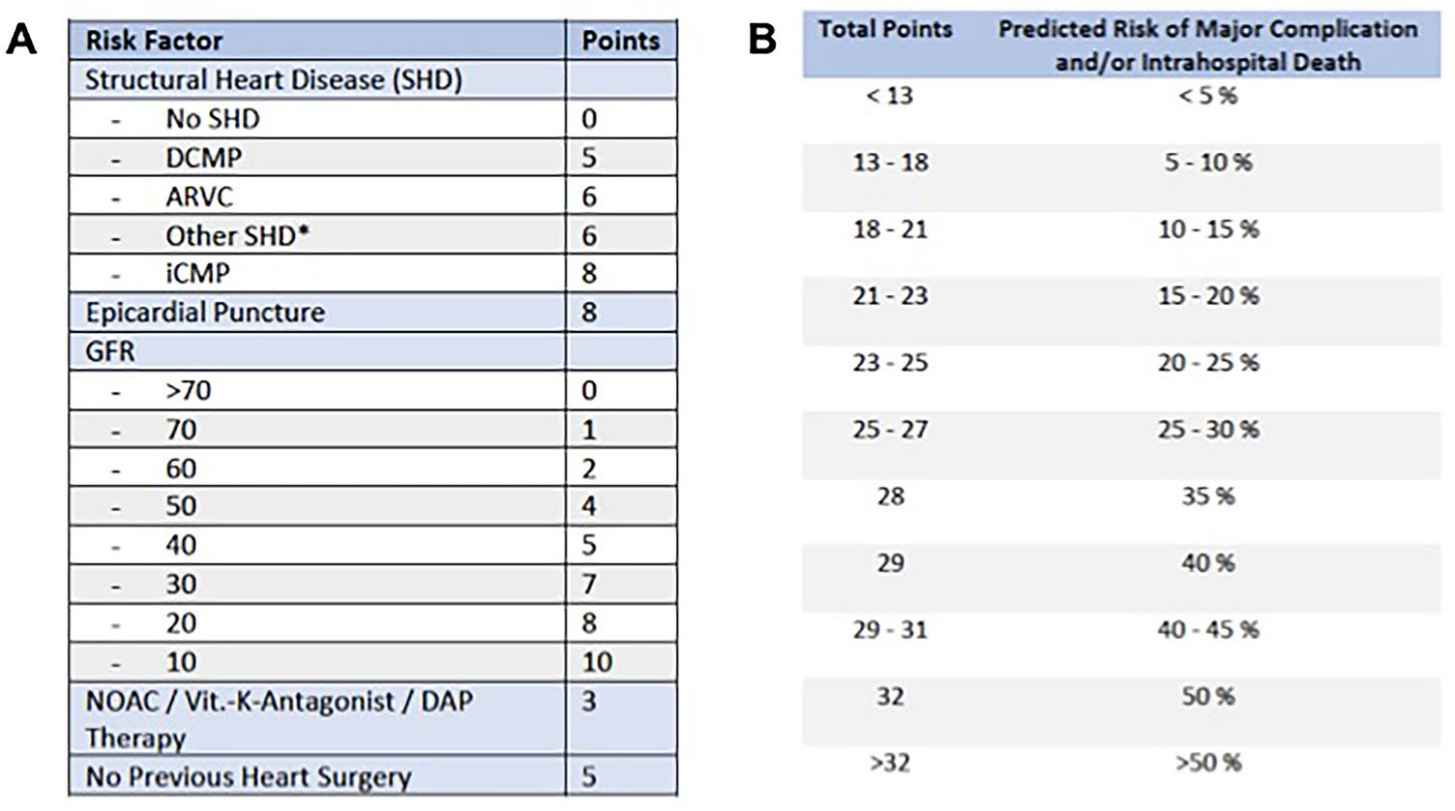
The RIVA score. **A** Calculates the points for each risk factor and **B** correlates the total score with the corresponding risk for major complications and/or intrahospital mortality. *ARVC *arrhythmogenic right ventricular cardiomyopathy, *DCMP *dilated cardiomyopathy, *iCMP *ischemic cardiomyopathy, *NOAC *novel oral anticoagulant, *DAP Therapy *dual antiplatelet therapy. *Including valvular heart disease, congenital heart disease, traumatic heart injury and patients with a history of myocarditis but preserved systolic left ventricular (LV) function

Based on the logistic regression analysis the risk in ventricular tachycardia ablation (RIVA) score was developed to allow prediction of a combined endpoint of major periprocedural complications and intrahospital death of patients undergoing VA ablation (Fig. 4, Supplemental Figure 1). The predictors are different entities of structural heart disease (iCMP, DCMP, ARVC, other and no SHD), previous heart surgery, OAC or antithrombotic therapy, epicardial ablation and glomerular filtration rate. The latter enters the model with a linear and non-linear part. A higher GFR is associated with a decreased risk of complications [GFR  =  80 compared to GFR  =  60, Odds ratio (95% confidence interval) 0.74 (0.60, 0.92)]. An epicardial ablation increases [Odds ratio 3.28 (2.00, 5.40)], a previous heart surgery reduces [Odds ratio 0.50 (0.27, 0.93)] and anticoagulation increases [Odds ratio 1.69 (1.04, 2.74)] the risk of complications. All forms of structural heart disease raise the risk of severe complications: Ischemic CMP [Odds ratio 3.29 (1.61, 6.72)], Dilated CMP [Odds ratio 2.12 (0.96, 4.69)], ARVC [Odds ratio 2.51 (0.92, 6.85)], Other SHD [Odds ratio 2.36 (0.93, 6.02)].

## Discussion

We report on procedural success, the occurrence of procedure-related complications and intrahospital death in a large patient population undergoing ablation of VA in an experienced high-volume center. The major findings of this study are (1) VA ablation is safe in patients without SHD and if only RV access is needed, (2) periprocedural complications and intrahospital death significantly increases in patients with SHD, (3) the type of access to the RV or LV significantly influences procedural safety and (4) presence of SHD, epicardial ablation, OAC or dual antiplatelet therapy are independent predictors of major complications and intrahospital death while a history of heart surgery predicts a lower risk for complications in ablation procedures. Therefore, the RIVA score using five standard parameters was developed to assess patient procedural risk.

### Safety of VA ablation procedures: influence of underlying heart disease

To our knowledge, this analysis reports the largest number of VA ablation procedures in idiopathic VT and patients with SHD so far. Patients with SHD had significantly more complications and a higher intrahospital mortality as compared to patients without underlying SHD undergoing VA ablation. This observation is in line with previous studies [5, 6, 16, 17]. Our findings are of importance since VA ablation in patients with SHD is often a pure symptomatic treatment and risk assessment has to be done by the treating physicians prior to the procedure. Our data show that VA ablation is a safe procedure with low rates of complications and a low intrahospital mortality in patients without SHD. The increase in procedural complications in patients with SHD may be due to the presence of comorbidities and the use of antiplatelet or OAC therapy. This is reflected by our findings that OAC and DAPT independently predicted complications and death.

### Safety of VA ablation procedures: influence of access type

VA ablation procedures have become a widely applied therapeutic treatment modality over the last 20 years. We found that procedural safety not only relies on patient characteristics, but also on the type of ablation procedure. Procedures involving only access to the RV had a low frequency of periprocedural complications or intrahospital death as compared to more complex procedures involving RV and LV access. The highest number of complications was noted in patients undergoing epicardial mapping and ablation. Additionally, we found epicardial ablation to be an independent predictor of complications or death. This finding allows for preprocedural risk stratification based on the anticipated type of access needed for successful VA ablation.

One reason for the strong correlation between complications to access types can be found in the observation that the most common major complication in our analysis was cardiac tamponade. This is in line with previous reports on VA ablation [7–9]. The method chosen to access the LV mainly contributes to development of this complication since involvement of transseptal and/or subxiphoidal puncture bear a natural risk to cause cardiac perforation. Other common adverse events were vascular injury at the groin access site or abdominal complications due to subxiphoidal puncture, which needed surgical repair. Remaining complications like thromboembolic events were rarely observed. Based on our data VA ablation appears safe in patients with RV origin or idiopathic VT. Therefore we would recommend that surgical backup is available in cases requiring LV access and especially during epicardial mapping and ablation, as recommended by the recent consensus statement of the HRS/EHRA/APHRS/LAHRS [10].

### VA ablation procedure-related mortality

Intrahospital death occurred in 1.8% of all VA procedures and was significantly more common in patients with SHD and in procedures with a LV arrhythmogenic substrate. A previous report described a mortality rate of 5% within 31 days after VA ablation in a large cohort of patients with SHD [11]. We found intrahospital death in 2.9% of all cases with SHD. Comparison between the above mentioned study and our results is difficult since we included only deaths which occurred during the hospital stay for VA ablation. Nevertheless, about half of the patients in the study by Santangeli et al. suffered from intrahospital mortality [11], resulting in comparable frequencies of intrahospital deaths in both studies.

We found four major causes for intrahospital mortality: cardiac tamponade, septic shock, cardiogenic shock and early VA recurrence. Santangeli found post-procedural VA recurrence as predictors of mortality [11]. This is in line with our observations that absence of procedural success predicted occurrence of complications and death and that early VA recurrence was a major cause of intrahospital death in our analysis. In addition, in-hospital death due to sepsis was a major cause of intrahospital death in our study. Of note, it occurred more frequently in patients with general anesthesia and endotracheal intubation, indicating that these patients could be at a late stage of their disease in our population.

### Implications for clinical practice: the RIVA score

Estimation of patient procedural risk is of importance before medical interventions. Several groups published risk scores which predict periprocedural adverse events and the success of VA ablation. The PAINESD score was developed based on a cohort of 193 patients to predict acute hemodynamic decompensation in patients undergoing VA ablation [12, 13]. This score was prospectively reevaluated by an independent multi-center study [14] and was adapted to predict periprocedural mortality of VA ablation in scar-related VT [11]. Vergara and colleagues developed another score to predict survival and freedom from arrhythmia recurrence after VT ablation using assessment of LV function and presence of cardiomyopathy, ICD or CRT and history of previous ablation [15]. So far there is no combined risk score, which allow to calculate the periprocedural complication rate and intrahospital mortality. Both parameters of individual risk stratification play a key role for the physician and the patient, when planning a VA procedure.

The present study developed a score based on the data of 1792 VA ablation procedures. Our data allow the formation of a simple score using five standard variables to predict probability of periprocedural complications or intrahospital death in patients scheduled for VA ablation. The score can be calculated in every patient before VA ablation using the patient history, standard laboratory measurements and ECG documentation to assess whether the clinical arrhythmia requires an epicardial ablation approach. This allows for a risk stratification of patients. There is no score enabling risk stratification in “all comers” of VA catheter ablation procedures to estimate the periprocedural risk. Previously developed scoring systems are preserved for patients with structural heart disease and scar-related VTs. We believe that the RIVA score can have major impact in planning future ablation procedures, for example to guide use of general anesthesia and presence of anesthesiologists during ablation procedures. A major strength of the RIVA score is the simplicity which underlies its estimation and the applicability of the score to all patients being scheduled for VA ablation procedures. However more studies are necessary to confirm the use of the score in prospective patient cohorts.

### Limitations

This was a retrospective study with its typical limitations. Our analysis contains only cases of intrahospital complications and mortality without analyzing the 30-day event rate. Further studies are necessary to prospectively evaluate the RIVA risk score. Nevertheless, our analysis is to our knowledge the largest study comparing VA ablation in patients with and without SHD and with detailed analysis of the various vascular and cardiac access types. The study periods spans over 15 years, including several changes in ablation technology and clinical guidelines limiting direct transfer of study results to current clinical practice. Information on procedures which were performed outside of our center were not available.

### Conclusion

Safety of VA catheter ablation mainly relies on patients baseline characteristics and the type of access into the ventricles, or epicardial space. VA ablation in patients without SHD and with pure RV involvement is safe, whereas procedural risk increases in patients with SHD and when LV access or epicardial ablation is necessary. The RIVA score encompassing of five variables allows for risk assessment of patients undergoing VA ablation.

## Supplementary Information

Below is the link to the electronic supplementary material.

Supplementary file 1 (DOCX 81 KB)

Supplementary file 2 (TIFF 5130 KB)

## Abbreviations

AAD: Anti-arrhythmic drug therapy
ARVC: Arrhythmogenic right ventricular cardiomyopathy
DAPT: Dual antiplatelet therapy
dCMP: Dilated cardiomyopathy
iCMP: Ischemic cardiomyopathy
NOAC: Novel oral anticoagulant
RV: Right ventricle
SR: Sinus rhythm
VA: Ventricular arrhythmia
VKA: Vitamin K antagonists
