# Supplementary material for: Complications and mortality after catheter ablation of ventricular arrhythmias: risk in VT ablation (RIVA) score
Source: Clin Res Cardiol. 2021 Jul 27;111(5):530–40. doi: 10.1007/s00392-021-01902-2 (PMC9054859; doi:10.1007/s00392-021-01902-2)
Supplement: Supplementary file 1 — (DOCX 81 KB) [file 392_2021_1902_MOESM1_ESM.docx]

# Supplemental Table 1: Overview of procedure related complications

| **N procedures** | 1792 |
| --- | --- |
| **N pts. with at least 1 major complication** | 77 (4.3) |
| **Tamponade (pericardiocentesis)** | 46 (3.0) |
| **Tamponade (surgical treatment)** | 10 (0.6) |
| **Aortic puncture with tamponade** | 3 (0.2) |
| **Abdominal organ perforation with surgery** | 7 (0.4) |
| **Stroke or TIA** | 5 (0.3) |
| **Systemic embolism** | 1 (0.1) |
| **Groin complication (surgical treatment)** | 8 (0.4) |
| **Procedure related MI / coronary vessel injury** | 6 (0.3) |
| **Peripheral vessel injury** | 1 (0.1) |
| **Pulmonary embolism** | 2 (0.1) |
| **PM lead dislogdement** | 1 (0.1) |
| **Intraprocedural CPR** | 1 (0.1) |
| **N pts. with at least 1 minor complication** | 71 (4.0) |
| **Minor groin complication** | 69 (3.9) |
| **Pneumothorax, conservative** | 4 (0.2) |

Values are N (%). SHD = structural heart disease, pts. = patients, RV = right ventricle, LV = left ventricle, TIA = transient ischemic attack PM = pacemaker, CPR = cardiopulmonary resuscitation.
