# Supplementary figures and images for: Complications and mortality after catheter ablation of ventricular arrhythmias: risk in VT ablation (RIVA) score
Source: Clin Res Cardiol. 2021 Jul 27;111(5):530–40. doi: 10.1007/s00392-021-01902-2 (PMC9054859; doi:10.1007/s00392-021-01902-2)

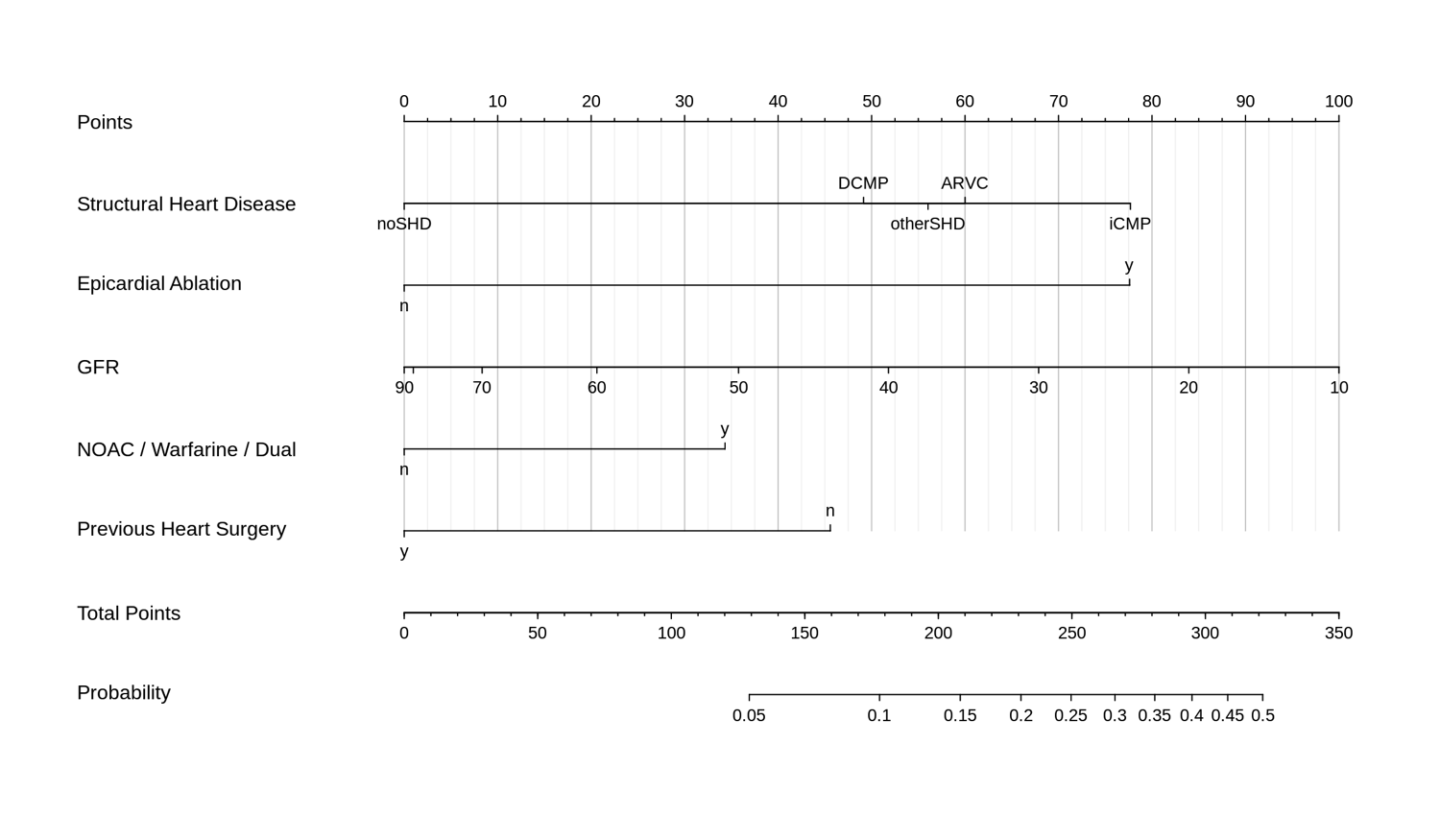

Supplement: Supplementary file 2 — (TIFF 5130 KB) [file 392_2021_1902_MOESM2_ESM.tiff]
